# Bilateral late hematomas after breast augmentation mimicking anaplastic large cell lymphoma: A case report

**DOI:** 10.1097/MD.0000000000036231

**Published:** 2023-12-01

**Authors:** I Zhen Ma, Jee Hyeok Chung, Jinhyun Kim, Ki Yong Hong

**Affiliations:** a Department of Plastic and Reconstructive Surgery, Seoul National University Hospital, Seoul National University College of Medicine, Seoul, Republic of Korea.

**Keywords:** BIA-ALCL, late hematoma, textured breast implant

## Abstract

**Rationale:**

Breast implant-associated anaplastic large cell lymphoma (BIA-ALCL) is rare, but its incidence has recently increased. It is characterized by a sudden onset of seroma collection after implantation of textured breast implants. However, BIA-ALCL may be confused with late hematoma, which is also a rare finding in aesthetic breast surgery. The cause of late hematoma is mostly unknown, and patients rarely present with specific symptoms.

**Patient concerns:**

We presented a case of late hematoma that occurred in a patient who underwent augmentation mammoplasty 25 years ago and was on anticoagulants for 7 years.

**Diagnoses:**

Ultrasonography and magnetic resonance imaging could not rule out the possibility of BIA-ALCL.

**Interventions:**

Bilateral implant removal was performed, and massive amounts of late hematoma and organizing tissues were removed.

**Outcomes:**

The pathologists confirmed the biopsy results as late hematoma with organizing tissues. Capsules from both sides were confirmed as fibrous capsules with chronic inflammation and foamy macrophage infiltration.

**Lessons:**

Although malignancy needs to be primarily ruled out, late hematoma can occur beyond expectations, especially in anticoagulated patients, and must be included in the differential diagnosis.

## 1. Introduction

Breast implant-associated anaplastic large cell lymphoma (BIA-ALCL) was first reported in 1997.^[1]^ Since then, awareness of this rare disease has increased. In 2016, the World Health Organization classified BIA-ALCL as a recognized entity.^[2]^ It is a distinct type of T-cell lymphoma that typically presents as a sudden collection of seroma or mass in the fibrous capsule surrounding the implant, after implantation of textured breast implants for reconstruction or for esthetic purposes.^[3]^ Although the exact incidence is unknown, it is estimated to be 2.03 per 1 million person-years and is on the rise.^[3]^

For patients suspected of BIA-ALCL, performing imaging studies such as ultrasonography or magnetic resonance imaging (MRI) for the evaluation of fluid collection, breast masses, and enlarged lymph nodes is recommended. If fluid collection is identified, fine-needle aspiration should be performed for cytological evaluation. A suspicious mass requires core-needle biopsy for pathologic workup.

In this article, we described a case of a patient who presented with bilateral breast swelling that had abruptly begun 4 years ago. Imaging studies showed signs of bilateral implant ruptures with complicated peri-implant fluid collection and nodular mass formation. BIA-ALCL was suspected, and implant removal was performed with subsequent multiple pathological evaluations.

## 2. Case

**Figure 1.**
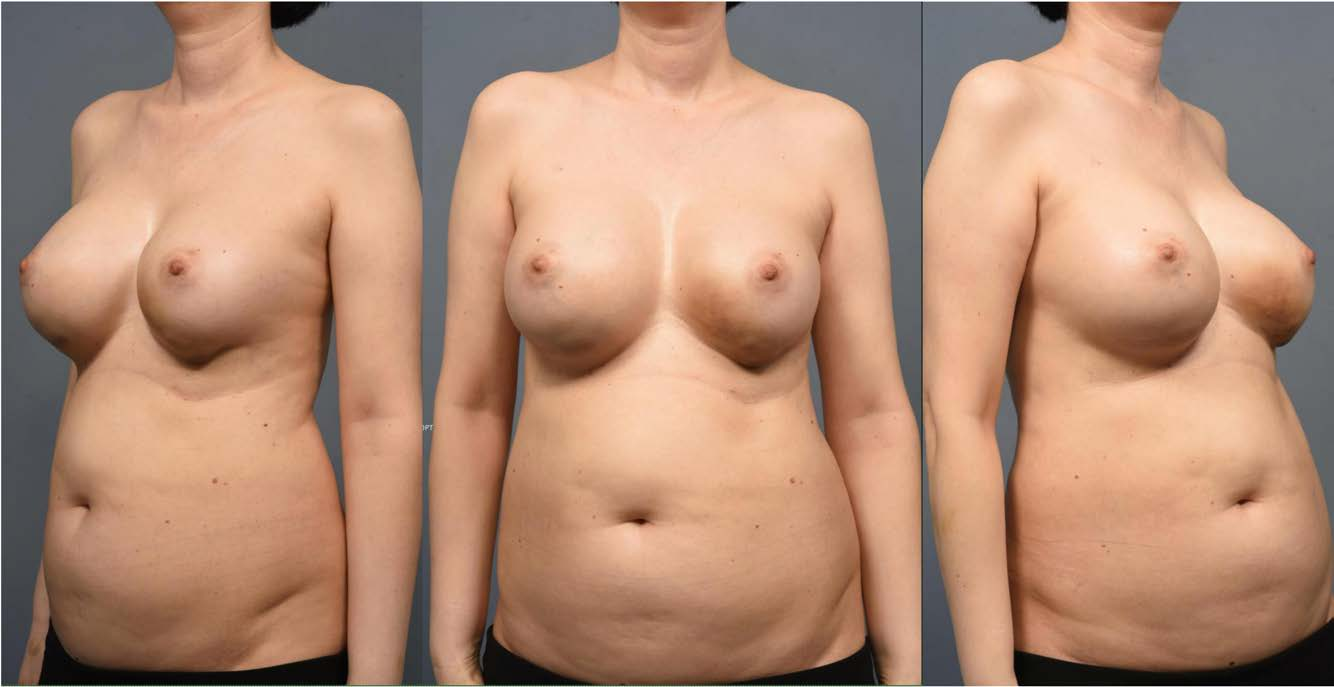
Preoperative photo of the patient. Preoperative photos of the patient showing bilateral breast swelling.

An informed consent was obtained from the patient regarding our study. This study was approved by the Institutional Review Board of our institution (IRB No. H-2000-000-000). A 56-year-old woman presented with bilateral breast swelling. Four years ago, an abrupt swelling occurred on the left breast, and a week later, the same symptom occurred on the right breast. According to the patient, several nodular lesions were also observed on both breasts because of the swelling (Fig. 1). She did not seek any medical advice previously, as the pain was tolerable.

The patient underwent bilateral augmentation mammoplasty 25 years ago at a local clinic, but did not recall the type or name of the inserted implants. She denied any history of trauma but had a middle cerebral artery infarction 7 years prior and had been taking aspirin and clopidogrel ever since. The swelling size gradually increased, and as the frequency and intensity of pain increased, she consulted our clinic for implant removal. On physical examination, nodular lesions were palpable on both sides near the inframammary fold, and skin thinning with a purplish color change at the lower pole of the left breast was observed.

**Figure 2.**
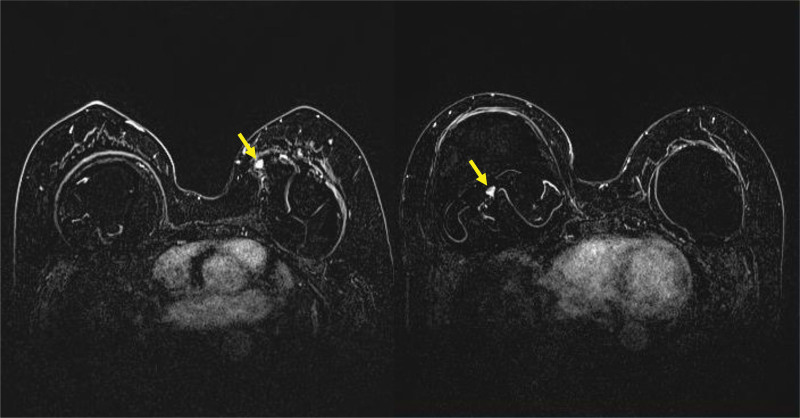
Breast magnetic resonance imaging (MRI). On MRI, fluid collection along with implant rupture on both sides was suspected. Several enhancing nodules (yellow arrows) were identified.

Based on the symptoms and physical examination results, we suspected BIA-ALCL. Ultrasonography revealed bilateral implant ruptures with fluid collection. Although fine-needle aspiration was scheduled, the patient wished for implant removal without regard to the results; therefore, aspiration was performed intraoperatively. MRI confirmed fluid collection and implant rupture on both sides. In addition, several enhancing nodules were identified on both sides of the fibrous capsule wall (Fig. 2). Biopsies were planned intraoperatively.

**Figure 3.**
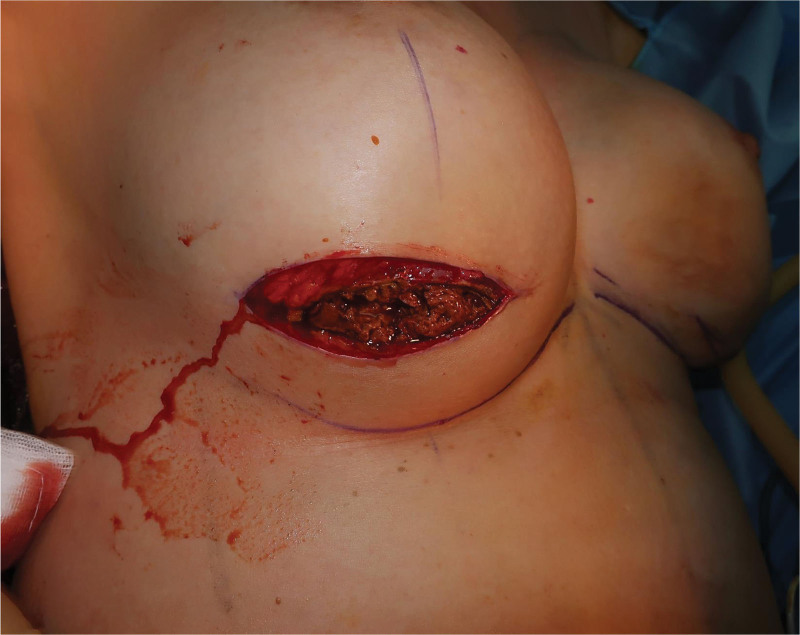
Peri-implant surrounding materials. Massive amounts of late hematoma and organizing tissues surrounding the implants.

**Figure 4.**
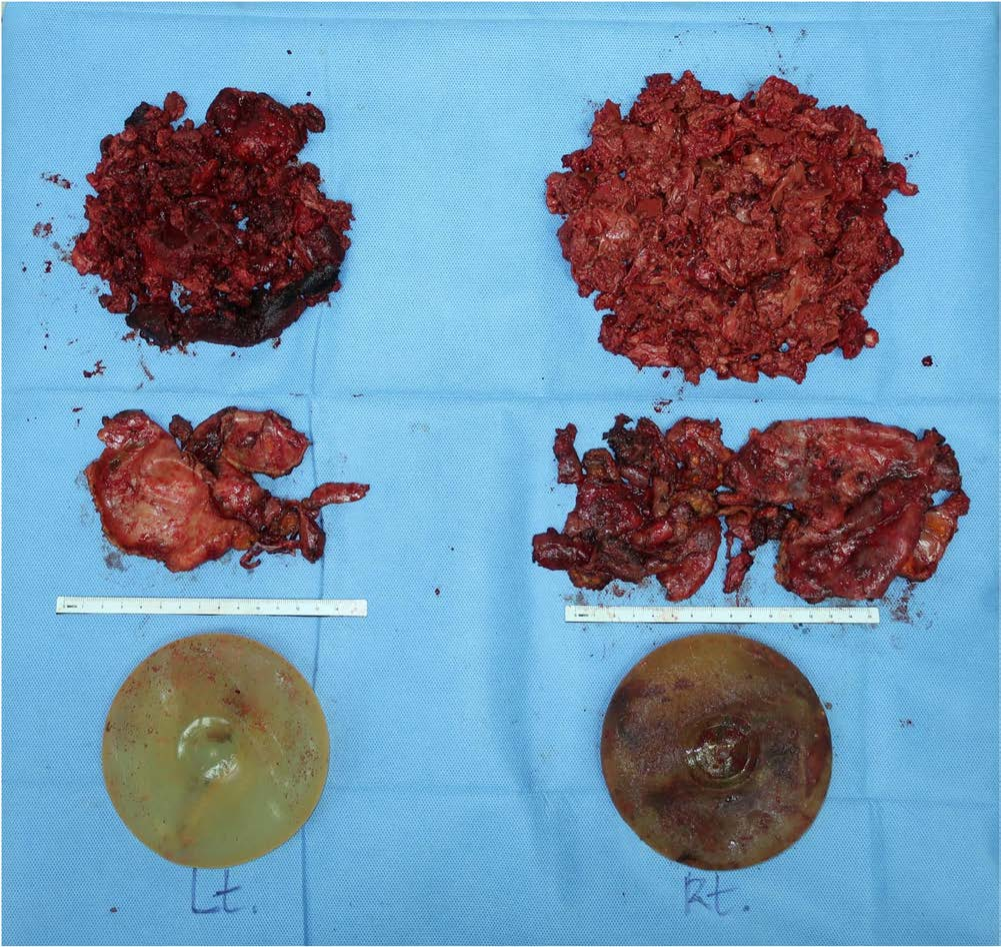
Specimen photo. (Top) Removed late hematoma from both sides. (Middle) Removed capsules. (Bottom) Removed breast implants.

**Figure 5.**
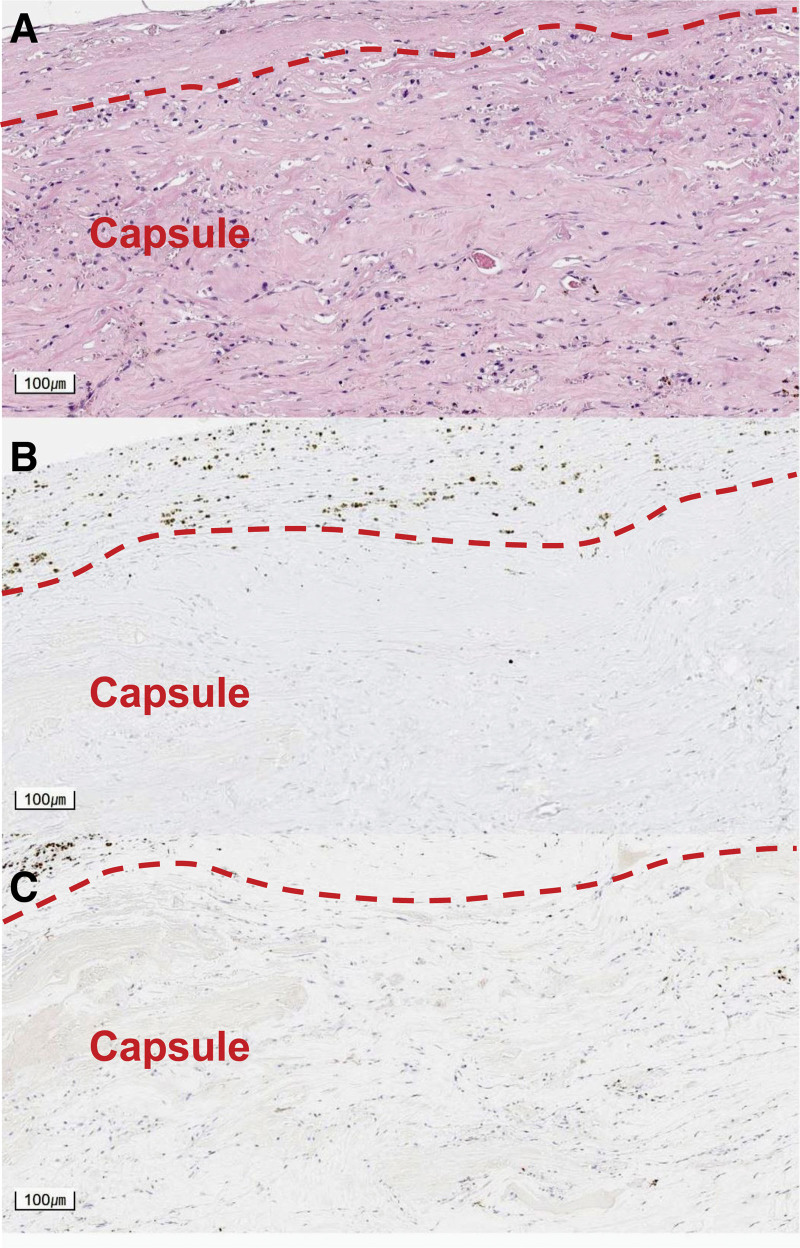
Histologic analyses of the specimens. Pathologic slide images of nodules within the capsule from right breast stained with hematoxylin and eosin (A), anaplastic lymphoma kinase (ALK) (B), CD30 (C) under the microscope in × 240 magnification. The present case showed negative results for ALK and CD30.

Under general anesthesia, a sanguineous turbid fluid spurted out from the puncture site upon aspiration. Implants were removed, and textured silicone implants were confirmed. The right implant was severely ruptured, with blood clots embedded inside the silicone contents. However, the left implant was intact and without any leakage. The implants were surrounded by massive amounts of pinkish-gray-colored old hematomas (Fig. 3). Over 500 cc of the material was removed from each side (Fig. 4). Upon observation of the late hematoma, our suspicion of BIA-ALCL had decreased, and therefore, frozen biopsy was not performed. For assurance, the removed specimens, including the area of MRI enhancement, were sent to pathology for BIA-ALCL evaluation. The capsules on both sides seemed normal, but as the removed implants had textured surfaces, total capsulectomy was performed. After the operation, the patient recovered without any significant complications. Cytology of the fluid did not show malignant cells. Additionally, immunohistochemistry (IHC) staining showed that CD30 and anaplastic lymphoma kinase (ALK) were negative (Fig. 5). Finally, the pathologists confirmed the biopsy results as late hematoma with organizing tissues. Capsules from both sides were confirmed as fibrous capsules with chronic inflammation and foamy macrophage infiltration.

## 3. Discussion

The most common presentation of patients with BIA-ALCL is sudden, spontaneous peri-implant fluid collection, leading to breast swelling. According to a study, 66% of patients diagnosed with BIA-ALCL were seen with seroma collection as the initial symptom, 8% with mass, and 7% with both seroma and mass. In addition, most patients had breast implant insertion approximately 10 years before diagnosis.^[3]^ The risk of BIA-ALCL is thought to increase over time, which is alarming to the population with textured implants who have yet to approach the 10-year time mark.^[4]^

Although the contents were unclear, our patient complained of fluid collection that had begun approximately 21 years after receiving bilateral augmentation mammoplasty. In addition, radiologic findings suggestive of bilateral implant ruptures with several enhancing nodules had grown our attention towards the diagnosis of BIA-ALCL.

Unlike other lymphomas, surgical excision is regarded as the first-line treatment for BIA-ALCL and has favorable outcomes in most patients. However, late diagnosis can lead to fatal conditions. Since the vast majority of plastic surgeons are unlikely to have encountered BIA-ALCL, it is important to include this rare entity in the differential diagnosis and perform work-ups whenever there is a possibility.^[3]^ Monitoring protocols were suggested, including radiologic studies such as ultrasonography and MRI, to better diagnose and manage patients suspected of BIA-ALCL.^[5]^ IHC staining of specimens is another tool to differentiate BIA-ALCL, as all of the pathologically confirmed BIA-ALCL cases were ALK-negative and positive for CD30 cell surface protein expression.^[6]^ Our case showed negative IHC staining results for ALK and CD30, confirming that it was not BIA-ALCL.

In the past, there have been a few cases of BIA-ALCL mimicking lesions that were finally diagnosed as late hematoma and silicone granuloma.^[7,8]^ Silicone granuloma almost always occur along with the rupture of implants. Risk factors for such silicone implant rupture include increased patient age, submuscular pocket placement of implants, and prolonged duration of implant placement.^[7]^ Since many of the symptoms and signs of silicone granuloma overlap with that of BIA-ALCL, it should be included for differential diagnosis whenever implant ruptures are present.

Late hematoma also needs to be considered in the differential diagnosis of fluid collection in previously augmented breasts. It has been reported that late hematomas can even occur 41 years after breast implant insertion.^[9]^ Most patients do not have specific symptoms other than breast swelling, which makes diagnosis more difficult.^[8]^

Previous studies have attempted to describe the mechanism of late hematoma formation. Wang et al reported that the textured surface of breast implants evokes an intense inflammatory response with increased vascularity, which persists for a longer period of time. This may eventually increase the risk of late-onset bleeding.^[10]^ Georgiade et al also proposed that the use of corticosteroids at the time of breast implant insertion may be responsible for erosion and subsequent hematoma formation, and Marques et al suggested contracted capsular microfracture as the cause of late-onset bleeding.^[11,12]^ Willens et al reported a patient who developed late intracapsular hematoma while being on anticoagulation 15 years after implant insertion.^[13]^

The patient in our case was also on anticoagulants for 7 years. Although the relationship between anticoagulation history and late hematoma formation is still unclear, spontaneous or trauma-induced hematoma may have been aggravated due to anticoagulation use.

The development of sudden fluid collection in patients with a history of long-standing breast implants leads to the possibility of BIA-ALCL. This is also true in cases where implant removal was performed, especially in cases where BIA-ALCL developed even 6 years after implant removal.^[3]^ In addition, physicians who encounter patients on anticoagulants with augmentation mammoplasty history must be aware of the possibility of late hematoma formation.

Despite the growing interest and increasing incidence of BIA-ALCL, the exact cause is yet to be proven. In patients with suspected BIA-ALCL, imaging studies, such as ultrasonography and MRI, are useful tools for diagnosis. However, they may not be able to exactly differentiate the possible fluid contents, such as seroma, hematoma, or ruptured implant materials. This case is meaningful in that late hematoma can occur beyond expectations, especially in anticoagulated patients. Although malignancy needs to be primarily ruled out, rarely occurring late hematoma also needs to be suspected, especially in patients who have an increased chance of bleeding.

## Author contributions

Conceptualization: Ki Yong Hong.

Investigation: I Zhen Ma, Jinhyun Kim, Jee Hyeok Chung.

Supervision: Jee Hyeok Chung, Ki Yong Hong.

Writing – original draft: I Zhen Ma, Jinhyun Kim.

Writing – review & editing: I Zhen Ma.

## References

[1] Keech JAK Jr Creech BJ. Anaplastic T-cell lymphoma in proximity to a saline-filled breast implant. Plast Reconstr Surg. 1997;100:554–5. 9252643 10.1097/00006534-199708000-00065

[2] Swerdlow SH Campo E Pileri SA. The 2016 revision of the World Health Organization classification of lymphoid neoplasms. Blood. 2016;127:2375–90. 26980727 10.1182/blood-2016-01-643569 PMC4874220

[3] Leberfinger AN Behar BJ Williams NC. Breast implant-associated anaplastic large cell lymphoma: a systematic review. JAMA Surg 2017;152:1161–8. 29049466 10.1001/jamasurg.2017.4026

[4] Nelson JA Dabic S Mehrara BJ. Breast implant-associated anaplastic large cell lymphoma incidence: determining an accurate risk. Ann Surg. 2020;272:403–9. 32694446 10.1097/SLA.0000000000004179 PMC8336676

[5] Santanelli di Pompeo F Laporta R Sorotos M. Breast implant-associated anaplastic large cell lymphoma: proposal for a monitoring protocol. Plast Reconstr Surg. 2015;136:144e–51e. 26218387 10.1097/PRS.0000000000001416

[6] Thienpaitoon P Disphanurat W Warnnissorn N. Breast implant-associated anaplastic large cell lymphoma in an Asian patient: the first case report from Thailand. Arch Plast Surg 2020;47:478–82. 32713177 10.5999/aps.2020.00108 PMC7520251

[7] Shepard E Kamenko S Snir OL. Silicone granuloma mimicking Breast Implant Associated Large Cell Lymphoma (BIA-ALCL): a case report. Case Reports Plast Surg Hand Surg 2020;7:63–7. 32596416 10.1080/23320885.2020.1762495 PMC7301711

[8] Fioramonti P Lovero S Kaciulyte J. An unusual case of late hematoma after implant-based breast reconstruction mimicking an anaplastic large cell lymphoma: a case report and literature review. Eur J Plast Surg 2021;45:187–96.

[9] Nahabedian MY. Explantation of 41-year-old implants following primary breast augmentation. Ann Plast Surg. 2007;58:91–4. 17197950 10.1097/01.sap.0000227520.00124.51

[10] Wang BH Chang BW Sargeant R. Late capsular hematoma after breast reconstruction with polyurethane-covered implants. Plast Reconstr Surg. 1997;102:450–2. 10.1097/00006534-199808000-00026 9703084

[11] Georgiade NG Serafin D Barwick W. Late development of hematoma around a breast implant, necessitating removal. Plast Reconstr Surg. 1979;64:708–10. 504495

[12] Marques AF Brenda E Saldiva PH. Capsular hematoma as a late complication in breast reconstruction with silicone gel prostheses. Plast Reconstr Surg. 1992;89:543–5. 1311114 10.1097/00006534-199203000-00026

[13] Willens HJ Wald H Kessler KM. Late intracapsular hemorrhage in an anticoagulated patient with a breast implant. Chest. 1996;110:304–5. 10.1378/chest.110.1.304-a 8681657

